# The external validation of a difficulty scoring system for predicting the risk of intraoperative complications during laparoscopic liver resection

**DOI:** 10.1186/s12893-019-0645-y

**Published:** 2019-11-27

**Authors:** Arpad Ivanecz, Irena Plahuta, Tomislav Magdalenić, Matej Mencinger, Iztok Peruš, Stojan Potrč, Bojan Krebs

**Affiliations:** 1 Department of Abdominal and General Surgery, University Medical Center Maribor, Ljubljanska 5, 2000 Maribor, Slovenia; 2 Faculty of Civil Engineering, Transportation Engineering and Architecture, University of Maribor, Smetanova ulica 17, 2000 Maribor, Slovenia; 3 Center of Applied Mathematics and Theoretical Physics, University of Maribor, Mladinska 3, 2000 Maribor, Slovenia; 4 Institute of Mathematics, Physics and Mechanics, Jadranska 19, 1000 Ljubljana, Slovenia; 5 Faculty of Natural Science and Engineering, University of Ljubljana, Aškerčeva cesta 12, 1000 Ljubljana, Slovenia

**Keywords:** Artificial neural network, Liver resection, Laparoscopy, Predictive score, Intraoperative complication, Cumulative distribution function, Mean risk curve

## Abstract

**Background:**

This study aimed to externally validate and upgrade the recent difficulty scoring system (DSS) proposed by Halls et al. to predict intraoperative complications (IOC) during laparoscopic liver resection (LLR).

**Methods:**

The DSS was validated in a cohort of 128 consecutive patients undergoing pure LLRs between 2008 and 2019 at a single tertiary referral center. The validated DSS includes four difficulty levels based on five risk factors (neoadjuvant chemotherapy, previous open liver resection, lesion type, lesion size and classification of resection). As established by the validated DSS, IOC was defined as excessive blood loss (> 775 mL), conversion to an open approach and unintentional damage to surrounding structures. Additionally, intra- and postoperative outcomes were compared according to the difficulty levels with usual statistic methods. The same five risk factors were used for validation done by linear and advanced nonlinear (artificial neural network) models. The study was supported by mathematical computations to obtain a mean risk curve predicting the probability of IOC for every difficulty score.

**Results:**

The difficulty level of LLR was rated as low, moderate, high and extremely high in 36 (28.1%), 63 (49.2%), 27 (21.1%) and 2 (1.6%) patients, respectively. IOC was present in 23 (17.9%) patients. Blood loss of >775 mL occurred in 8 (6.2%) patients. Conversion to open approach was required in 18 (14.0%) patients. No patients suffered from unintentional damage to surrounding structures. Rates of IOC (0, 9.5, 55.5 and 100%) increased gradually with statistically significant value among difficulty levels (*P* < 0.001). The relations between the difficulty level, need for transfusion, operative time, hepatic pedicle clamping, and major postoperative morbidity were statistically significant (*P* < 0.05). Linear and nonlinear validation models showed a strong correlation (correlation coefficients 0.914 and 0.948, respectively) with the validated DSS. The Weibull cumulative distribution function was used for predicting the mean risk probability curve of IOC.

**Conclusion:**

This external validation proved this DSS based on patient’s, tumor and surgical factors enables us to estimate the risk of intra- and postoperative complications. A surgeon should be aware of an increased risk of complications before starting with more complex procedures.

## Background

Since the first report of laparoscopic liver resection (LLR) in 1991, the laparoscopic feasibility of all liver resections has been demonstrated [1–3]. The use of minimally invasive liver surgery has been supported by consensus conferences in 2008 and 2014 [4, 5]. Recently, the number of LLRs performed worldwide has increased exponentially [6]. Moreover, its benefits over traditional open liver surgery have been proved by prospective randomized trials [7, 8].

However, population-based studies show LLR is still limited to a few specialists in tertiary liver centers [9]. The technical complexity of procedures namely varies from peripheral wedge resections to major hepatectomies and a considerable learning curve must be overcome [10, 11]. The European Guidelines Meeting for Laparoscopic Liver Surgery in 2017 highlighted the need for a stepwise progression through the learning curve to minimize morbidity [12]. Therefore, preoperative assessment of the difficulty of LLR is important in selecting appropriate patients according to a surgeon’s skills and experience at each stage of the learning curve [12].

Different scores have been proposed to rate the difficulty of LLR and the need for validations of existing tools before the clinical application has been highlighted [13–16]. Some of the proposed surgical difficulty scoring systems (DSS) have been subjected to several external validations [17–25]. Recently, Halls et al. used a large multicenter European database to develop and internally validate a DSS estimating the risk of intraoperative complications (IOC) during LLR [26]. To our knowledge, it has not been externally validated to date.

The study aims to externally validate this DSS [26] in terms of the original outcome – IOC. Furthermore, some intra- and postoperative outcomes are going to be studied according to the proposed difficulty levels.

## Patients and methods

### The aim of the study and patients

This study aimed to externally validate the DSS by Halls et all [26]. and to upgrade it by proposing the risk curve for predicting the probability of IOC.

A retrospective review of a prospectively maintained database of patients who underwent liver surgery at the Department of Abdominal and General Surgery of University Medical Center Maribor in Slovenia was performed. This is a specialized referral center for hepato-pancreato-biliary surgery.

The study period lasts from April 2008 until 28 February 2019. The study was based on the intention-to-treat principle. Consecutive patients undergoing a planned pure LLR were chosen. Liver resection planned to be completed laparoscopically was included, except cyst fenestration, liver biopsies, and radiofrequency ablation. Short-term outcomes of several patients from this cohort have been published previously [27–29].

Several routinely available variables were reviewed from the database and analyzed since the patients underwent routine diagnostic workup consisted of blood count, chest radiography, abdominal computed tomographic scans with contrast enhancement, and/or liver-specific contrast magnetic resonance imaging. Their preoperative liver function was assessed according to the Child-Pugh classification [30]. In some cases, the indocyanine green retention test and computed tomographic volumetric analysis were needed.

The indications for LLR were the same as for open liver resections. Contraindications for the laparoscopic approach were modified over time. The absolute contraindications included the need for biliary or vascular resection and reconstruction, the need for multi-visceral en-bloc resection and resections for hilar cholangiocarcinoma [26].

All patients were operated on by the same surgeon (AI) who is responsible for a laparoscopic liver program. Only pure LLR has been performed, no hand-assisted or hybrid procedures were used. Surgical techniques were applied as reported [27]. Briefly, patients were placed in the supine position, except for the resection of posterosuperior segments of the liver when the left lateral decubitus position was used. The placement of trocars was based on tumor location. Laparoscopic ultrasonography of the liver was mandatory. A pneumoperitoneum of 12–14 mmHg and a central venous pressure less than 5 cm H_2_O was maintained during hepatic parenchymal transection. A hepatic pedicle clamping (Pringle’s maneuver) was applied selectively and intermittently, following the rule of 15-min clamp and 5-min release. For the hepatic parenchymal transections different high energy devices (a cavitron ultrasonic surgical aspirator, harmonic scalpel or an electrothermal bipolar tissue sealing system) were used. Larger structures were controlled with endoclips. Endoscopic linear stapler devices were used for transection of large pedicles and hepatic veins. The resected liver specimen was placed in a plastic bag and removed through an enlarged port site or through a suprapubic Pfannenstiel incision without muscular section.

Patients had given consent that anonymous data can be used for research purposes at the time of the operation. Their records were anonymized and de-identified before analysis. Ethical approval for this study was obtained from the institutional review board.

### Data collection and definitions

Basic patient demographics and clinical factors were examined. It included age, sex, body mass index, performance status defined according to the American Society of Anesthesiologists (ASA) score and presence of liver cirrhosis (Child-Pugh score/grade).

For the requirements of the study, neoadjuvant chemotherapy, tumor characteristics (malignant or benign, its size), and previous abdominal surgical history were examined. The location of the tumor was defined as anterolateral in segments 2, 3, 4b, 5, 6 and posterosuperior in segments 1, 4a, 7, and 8 [31]. The type of resection was categorized into three groups (minor, technically major, and anatomically major) [17]. Anatomically major resections involve 3 or more adjacent liver segments. Technically major resections are those that would be considered minor resections anatomically but are located in posterosuperior liver segments that are difficult to access laparoscopically [12].

### External validation of the difficulty scoring system and a mean risk curve

The DSS of LLR introduced by Halls et al. [26] was used and externally validated. Its parameters (neoadjuvant chemotherapy, previous open liver resection, benign or malignant lesion, lesion size and classification of resection) were captured from the institutional database. Each LLR was retrospectively scored from 0 to 15. Scores of 0–2, 3–5, 6–9 and 10–15 were then translated into respective difficulty levels: low, moderate, high, and extremely high. On the base of this scoring, these difficulty levels predict the likelihood of IOC as follows: < 10% for low, 10–20% for moderate, 20–50% for high and > 50% for extremely high levels [26] (Table 1).

**Table 1:** Difficulty scoring system by Halls et al. [26] at a glance

Risk factor and assigned points				
Risk factor	Risk factor category	Points assigned		
*Neoadjuvant chemotherapy*	No neoadjuvant chemotherapy	0		
	Received	1		
*Previous open liver resection*	No	0		
	Yes	5		
*Lesion type*	Benign	0		
	Malignant	2		
*Lesion size (cm)*	< 3	0		
	3–5	2		
	> 5	3		
*Classification of resection*	Minor	0		
	Technically major	2		
	Anatomically major	4		
Points translated into difficulty scores				
*Difficulty scores*	0–2	3–5	6–9	10–15
*Difficulty levels*	Low	Moderate	High	Extremely high
*Probability of IOC*	< 10%	10–20%	20–50%	> 50%

*IOC* intraoperative complication

As a primary validator was used IOC, described as an objective marker of a complex operation [26]. Key markers of IOC were blood loss over 775 mL, unintentional damage to the surrounding structures and conversion to open approach [26]. The conversion was defined as the requirement for laparotomy at any time of the procedure, except for the extraction of the resected specimen, because no hand-assisted or hybrid procedures were used [26].

Established surrogates of the technical difficulty, namely blood transfusion requirements, operative time, the need for the hepatic pedicle clamping and its duration were used as secondary validators. Postoperative morbidity was graded according to the Clavien-Dindo classification [32] and used as a secondary validator. Grades from 3a to 4b represent a major complication requiring invasive intervention and the use of organ support [32].

Two original models were built for the external validation: a linear model and a model based on a conditional average estimator artificial neural network (CAE ANN) [33, 34].

To obtain the mean risk curve for predicting the probability of IOC, Weibull cumulative distribution function (CDF) [35] and the Kolmogorov-Smirnov test [36] were used.

### Statistical analysis

The IBM SPSS for Windows Version 21.0 (IBM Corp., Armonk, NY, USA) was used for basic statistical analysis. Univariable analysis for binary data was performed using the chi-square test for categorical variables. The contingency table chi-square tests were performed for *α* = 0.05 and the *P*-value for the null hypothesis of no relationship between groups is present. The analysis of variance (ANOVA) was used to determine statistically significant differences among the means of three independent groups. The *P*-values are related to the one-way ANOVA test with the null hypothesis that the means of the groups are equal. There are only 2 patients in the category »Extremely high«, therefore all corresponding chi-square and ANOVA tests considered just the first three groups.

Wolfram Mathematica for Windows Version 10.4 (Wolfram Research, Inc., Champaign, IL, USA) was used for statistical computations and basic validation of the linear DSS introduced by Halls et al. [26]. Multivariate analysis of the data was performed by *LinearModelFit* command of Mathematica. The correlation between the independent variables was analyzed by the command *Correlation* of Mathematica. Percentages are reported at 1 decimal place, coefficients of the multivariate analysis are presented at 3 decimal places and the *P*-value < 0.05 was considered statistically significant. In addition, a CAE ANN [33, 34] was used as a statistical tool for nonlinear regression.

To determine the risk of IOC and obtain the continuous (theoretical) risk curve, the original data was tested to CDF for the Weibull [35] distribution ${y}_W=1-{e}^{-{\left(\frac{x}{\lambda}\right)}^k}$. Values of λ and *k* were calculated using the *FindFit* command of Mathematica.

The Kolmogorov-Smirnov test [36] tests is a nonparametric test that tests whether the given data originates from a proposed (i.e. Weibull) distribution. Testing was performed by using the command *DistributionFitTest* of Mathematica. The test statistics are defined in terms of a CDF of the hypothesized (in this case the Weibull) distribution. The Kolmogorov-Smirnov statistics represent the supremum distance between the hypothesized CDF and the CDF of the sample. The closer this number is to zero the more likely it is that the sample was drawn from the hypothesized distribution.

## Results

### Study population

From April 2008 to February 2019, a total of 128 consecutive patients underwent pure LLR and were enrolled in the study. The baseline characteristics of the patients are as follows: the average age was 63 (20–86) years, 76 (59.4%) patients were male, the average body mass index was 26.7 (18.0–50.1) kg/m^2^. ASA fitness grade distribution of patients was as follows: 33 (25.8%) of ASA I, 56 (43.8%) of ASA II, 36 (28.1%) of ASA III and 3 (2.3%) patients of ASA IV. Liver cirrhosis Child-Pugh A or B was present in 25 (19.5%) patients.

Indications for liver resection were malignant disease in 89 (69.5%) patients; namely colorectal liver metastases in 42 (32.8%), hepatocellular carcinoma in 28 (21.9%), intrahepatic cholangiocarcinoma in 11 (8.6%), and other types of malignancy in 8 (6.2%). Other variables, expressed as risk factors, are shown in Table 2.

**Table 2:** Risk factors for the intraoperative complication, assigned points and statistical analysis [26]

Risk factors	Overall	Points assigned	Intraoperative complication		*P*-value^a^
			Yes	No	
Number of patients	128	/	23 (17.9%)	105 (82.1%)	/
Neoadjuvant chemotherapy	22 (17.2%)	1	7	15	0.073
Previous open liver resection	2 (1.6%)	5	1	1	0.328
Malignant lesion	89 (69.5%)	2	22	67	0.003
*Lesion size*					
Lesion size < 3 cm	48 (37.5%)	0	1	47	0.938
Lesion size 3–5 cm	44 (34.4%)	2	9	35	0.596
Lesion size >5 cm	36 (28.1%)	3	13	23	0.001
*Classification of laparoscopic liver resection*					
Minor	93 (72.6%)	0	9	84	< 0.001
Technically major	18 (14.1%)	2	6	12	0.094
Anatomically major	17 (13.3%)	4	8	9	0.003

^a^Chi-square test

### Basic validation of a difficulty scoring system

The same five risk factors predicting IOC as proposed by Halls et al. [26] and their assigned points were included in an initial analysis. The results are presented in Table 2.

The statistical significance for IOC was reached for neoadjuvant chemotherapy, lesion type, lesion size > 5 cm and classification of resection, but not for previous open liver resection.

The LLR difficulty scores (DS) were calculated for every patient. Based on the score, patients were divided into four risk groups to estimate the risk of IOC as a primary validator. The surrogates of technical operative difficulty were analyzed as secondary validators. Grouping of patients into four difficulty levels and outcomes are shown in Table 3.

**Table 3:** Grouping of patients into difficulty levels and outcomes

	Overall *N* (%)	Low (0–2)	Moderate (3–5)	High (6–9)	Extremely high (10–15)	*P*-value^a^
Number of patients	128	36 (28.1%)	63 (49.2%)	27 (21.1%)	2 (1.6%)	/
Intraoperative complication [26]	23 (17.9%)	0 (0%)	6 (9.5%)	15 (55.5%)	2 (100%)	< 0.001
Transfusion required	17 (13.2%)	1 (2.7%)	11 (17.5%)	4 (14.8%)	1 (50.0%)	0.042
Operative time (min)^*^	155 (25–360)	120 (45–240)	150 (25–360)	210 (120–350)	310 (260–360)	< 0.001
Hepatic pedicle clamping	30 (23.4%)	2 (5.6%)	19 (30.2%)	9 (33.3%)	0 (0.0%)	0.009
Total hepatic pedicle clamping (min)^*^	30 (10–75)	37.5 (35–40)	30 (10–75)	40 (10–60)	0 (0–0)	0.033
Postoperative morbidity (Clavien-Dindo 3a ≤ 4b) [32]	12 (9.3%)	1 (2.7%)	4 (6.3%)	6 (22.2%)	1 (50.0%)	0.028

^*^Continuous variables are reported as median (range); ANOVA test. ^a^*P*-values were calculated by chi-square test or ANOVA, in both cases without considering the extremely high-risk group

The difficulty level of LLR was rated as low, moderate, high and extremely high in 36 (28.1%), 63 (49.2%), 27 (21.1%) and 2 (1.6%) patients, respectively. IOC was present in 23 (17.9%) patients. The median blood loss was 110 mL (range 0 to 2200 mL). Blood loss of >775 mL occurred in 8 (6.2%) patients. The conversion was required in 18 (14.0%) patients, but in none cases due to life-threatening bleeding. The need for conversion included unfavorable intra-operative findings ((inability to proceed due to dense adhesions (*n* = 2), difficult exposure of large, fatty liver (*n* = 2), inability to locate the tumor (*n* = 1), and slow progression of liver transection (*n* = 2)) or events (oncological concern due to uncertain localization of tumor margins (*n* = 9), need for diaphragm resection to assure radical resection (*n* = 1), and diffuse parenchymal bleeding (*n* = 1)). There has been no unintentional damage to the surrounding structures in any of the patients.

The rates of IOC (0, 9.5, 55.5 and 100%) increased gradually with statistically significant values among difficulty levels (*P* < 0.001). The rate of complications in the high-risk group (55.5% vs. 20–50%) slightly exceeded the proposed value.

Analysis of surrogate outcomes showed that transfusion was required in 17 (13.2%) patients. The median operative time was 155 min (range 25 to 360 min). Pedicle clamping was used in 30 (23.4%) patients. Total hepatic pedicle clamping time was 30 min (range 10 to 75 min). *P*-value among difficulty levels was < 0.05.

90-day major morbidity (Clavien-Dindo grades from 3a to 4b) occurred in 12 (9.4%) patients. Seven patients experienced grade 3a complications and were treated successfully by percutaneous drainage of pleural effusions and bile collections. Four patients required reoperations (grade 3b complication). Indications were postoperative bleeding from the port site (*n* = 1), anastomotic leakage from colorectal anastomosis after simultaneous laparoscopic liver and colorectal surgery (*n* = 1), port site omental protrusion (*n* = 1) and biliary leak with diffuse biliary peritonitis (*n* = 1). One cirrhotic (Child-Pugh A) patient who underwent resection of HCC experienced grade 4b complication with multi-organ dysfunction and prolonged intensive care unit admission.

The rates of postoperative complications (2.7, 6.3, 22.2 and 50%) increased gradually with statistically significant values among difficulty levels (*P* < 0.001).

The mortality rate was 0.8% with one postoperative death within 90 days. The patient (moderate difficulty level) had an alcoholic liver cirrhosis Child-Pugh B and died on a postoperative day 10 because of an unstoppable bleeding from ruptured esophageal varices.

### Multivariate linear and CAE ANN-based validation of a difficulty scoring system

An original data set consisting of a cohort of 128 patients were used for validating the 15 points criteria (Table 1) for predicting the risk of IOC as introduced by Halls et al. [26]. The dependent variable (*y*) is the 0–15 points risk prediction of IOC during LLR. The independent variables were considered as follows: *x*_1_ (neoadjuvant chemotherapy), *x*_2_ (previous open liver resection), *x*_3_ (lesion type), *x*_4_ (lesion size), and *x*_5_ (classification of resection).

The correlation between independent variables was found to be very weak (in the absolute range from 0.005 to 0.321). The *P*-values (for the null hypothesis that the corresponding coefficient is equal to zero) for the linear model

1 $$ y=-1.85180+0.67232{x}_1+1.48669{x}_2+2.12691{x}_3+0.35112{x}_4+1.88364{x}_5 $$

were found to be all < 0.01 (the highest P-value 0.002 was found for variable *x*_1_) . Standard errors for the intercept and the coefficients of *x*_1_, …, *x*_5_ are found to be 0.258, 0.213, 0.435, 0.201, 0.033, 0.126, respectively.

For any patient from our cohort, we denote the 15-score-value, *y*_*AL*_, obtained by the proposed linear model defined by eq. (1). It was compared to the scoring introduced by Halls et al. [26], denoted by *y*_*H*_. The linear (Pearson) correlation coefficient between *y*_*AL*_ and *y*_*H*_ was found to be very strong (0.914).

**Fig. 1:**
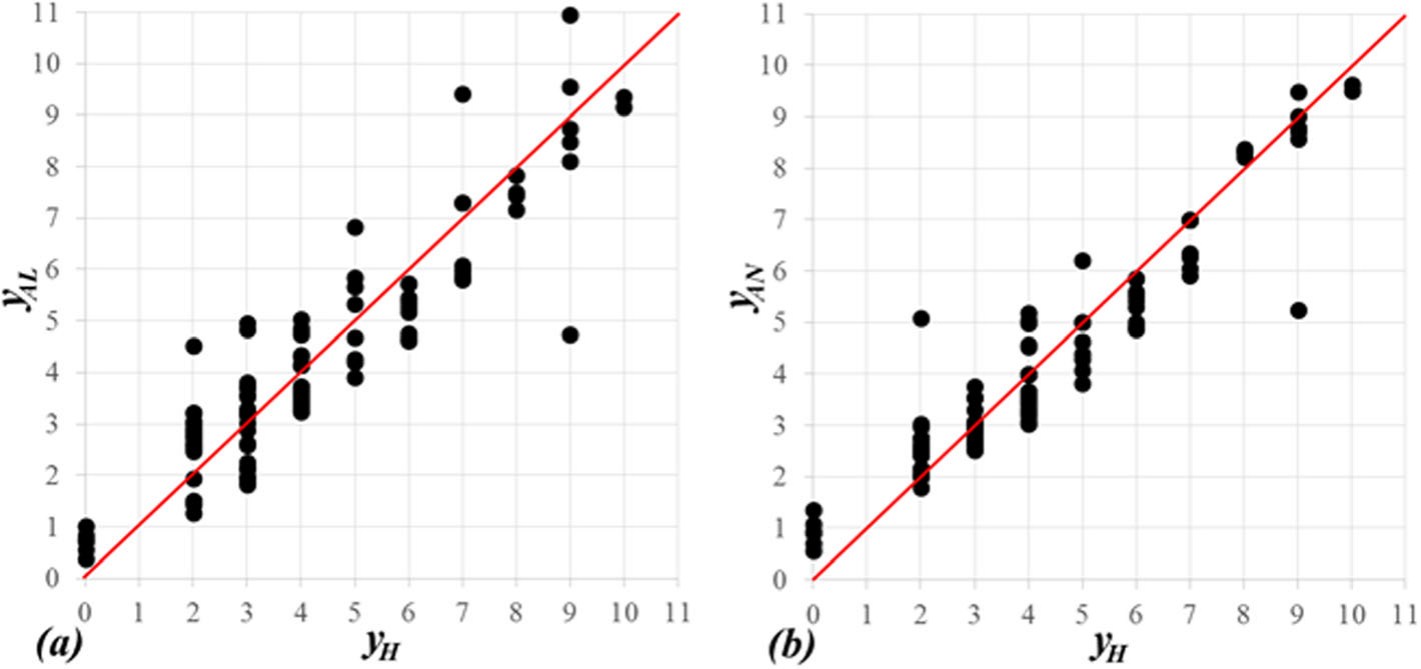
Correlation between DSS [26] and (**a**) linear as well as (**b**) nonlinear models, respectively. The regression lines with no intercepts: (a) *y*_*AL*_ = 0.95937*y*_*H*_ and (b) *y*_*AN*_ = 0.95913*y*_*H*_, respectively. The correlation coefficients: (a) 0.914 (b) 0.948

Linear relations of the form *y*_*AL*_ = *ky*_*H*_ and *y*_*AL*_ = *ay*_*H*_ + *b* were found to be *y*_*AL*_ = 0.95937*y*_*H*_ and *y*_*AL*_ = 0.83530*y*_*H*_ + 0.64983, respectively, with *P*-value < 0.001 for all coefficients. The scoring according to the proposed linear model vs. the scoring introduced by Halls et al. [26] (at the abscissa) are presented in Fig. 1 (a). The range of the data considered in the present study is 0 − 10. The reason is that only two patients in our cohort had previous open liver resection (see Table 2), and this variable has the highest score (five).

**Fig. 2:**
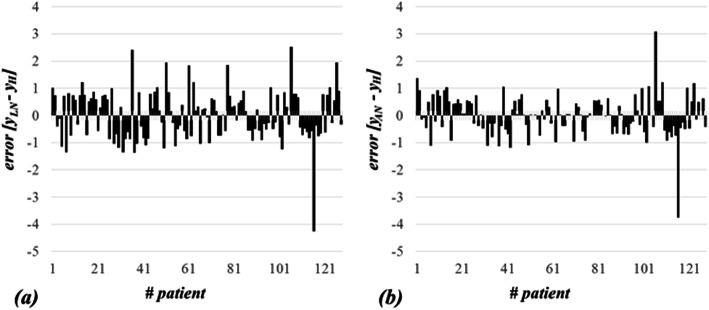
The graph of residuals for all 128 patients. The residuals are representing the discrepancy between original scoring by Halls et al. (*y*_*H*_) and our linear and nonlinear models denoted by *y*_*AL*_ and *y*_*AN*_, respectively. The maximal discrepancy for linear model (**a**), *y*_*AL*_ − *y*_*H*_, was found to be − 4.27 and for nonlinear model (**b**), *y*_*AN*_ − *y*_*H*_, was found to be − 3.76. In both cases the mean error is close to zero, which confirms high matching of models

Note that the coefficient of determination *R*^2^ for the linear model was found to be 0.83531, the mode (commonest) of the errors was found to be 0.86101, the median of the errors was found to be 0.09551, the (arithmetic) mean of the errors was found to be −0.00002. The graph of residuals for all 128 patients is presented in Fig. 2 (a).

Furthermore, the same procedure as described above was performed by CAE ANN. The 15-score value obtained by the proposed nonlinear model obtained by CAE ANN is denoted by *y*_*AN*_.

The linear correlation (Pearson coefficient) between *y*_*AN*_ and *y*_*H*_ was found to be very strong (0.948263). Linear relations of the form *y*_*AN*_ = *ky*_*H*_ and *y*_*AN*_ = *ay*_*H*_ + *b* were found to be *y*_*AN*_ = 0.95913*y*_*H*_ and *y*_*AN*_ = 0.84079*y*_*H*_ + 0.61983, respectively, with *P* value < 0.001. The scoring according to the proposed linear model vs. the scoring according to Halls et al. [26] (at the abscissa) are presented in Fig. 1(b).

Note that the coefficient of determination *R*^2^ for the nonlinear model was found to be 0.97443, the mode (commonest) of the errors was found to be −0.16540, the median of the errors was found to be 0.15560, the (arithmetic) mean of the errors was found to be 0.152934. The graph of residuals for all 128 patients is presented in Fig. 2 (b).

In this research the CAE ANN was used as a statistical tool for nonlinear regression. Basically, the procedure of estimating the nonlinear regression consists of two (independent) numerical parts. The first part corresponds to the self-organization of the artificial neurons (storing empirical information) and describes the observed phenomenon (i.e. observed Halls et al. score), while the second part corresponds to the optimal estimation of unknown parameters of the same phenomenon. Both parts are essential for automatic modeling of various (natural) phenomena [34, 37].

### The mean risk curve for predicting the probability of intraoperative complications

**Fig. 3:**
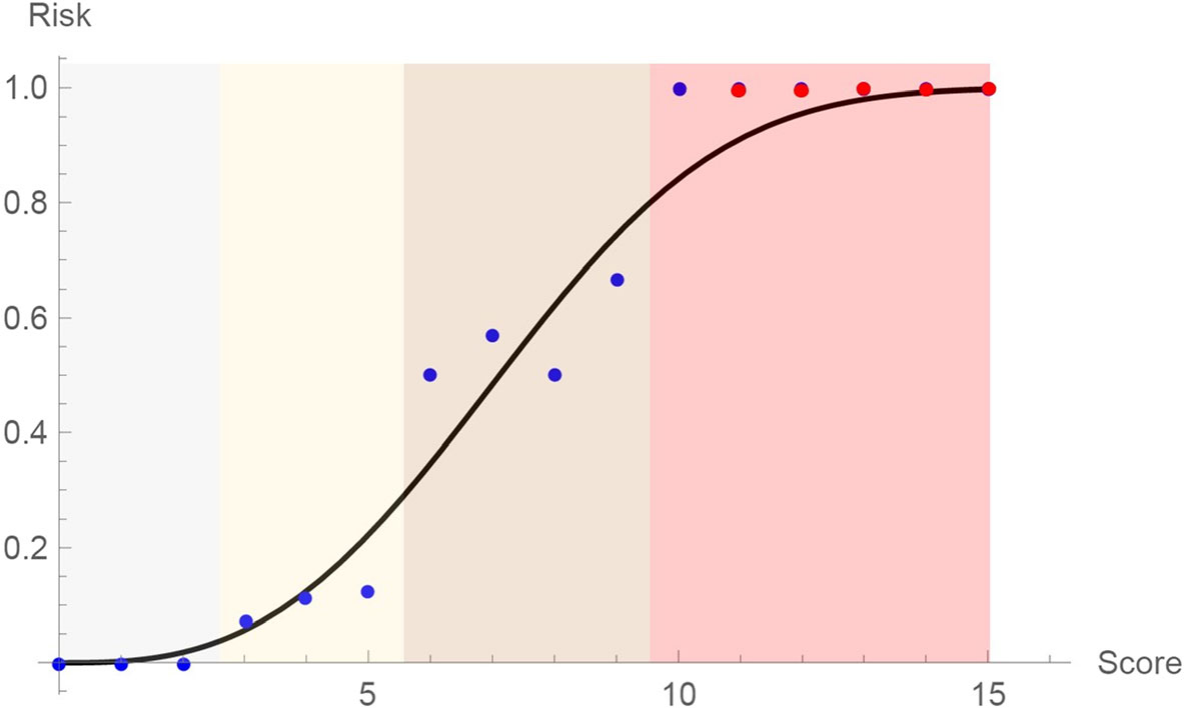
The continuous mean risk curve of IOC as a function of the DS. The blue dots are representing the discrete data from Table 4. The red dots are representing the assumed values. The solid black curve depicts a corresponding Weibull CDF [42], which represents the continuous mean risk curve of IOC. Different background colors are denoting the four-level scale (Low, Moderate, High, Extremely high).IOC, intraoperative complication; DS, difficulty score; CDF, cumulative distribution function

To determine the risk of IOC and obtain the continuous (theoretical) risk curve, the original data (see the blue dots in Fig. 3) was tested to CDF for the Weibull [35] distribution ${y}_W=1-{e}^{-{\left(\frac{x}{\lambda}\right)}^k}$. Using *FindFit* command of Mathematica, values of *λ* = 8.085 and *k* = 2.871 were calculated. The discrete results are presented in Table 4.

**Table 4:** The risk of the intraoperative complication for each difficulty score

DS	0–2	3	4	5	6	7	8	9	10–15
Risk of IOC	0%	7%	11%	13%	50%	57%	50%	67%	100%

The Kolmogorov-Smirnov statistics and the corresponding *P*-value were found to be 0.375, and 0.215, respectively. At the level of significance of 0.05, we cannot reject the null hypothesis that the sample was drawn from the Weibull distribution with parameters *λ* = 8.085 and *k* = 2.871. The graphical results are presented in Fig. 3.

## Discussion

During LLR, surgeons face difficulties due to patient, tumor, and surgery-related factors [26]. Classification systems for assessing the surgical difficulty of LLR have been proposed because they have turned qualitative judgments into quantitative assessments [13–16]. Recently, Halls et al. developed and internally validate a new DSS (Table 1) [26]. The present study was designed specifically to externally validate it [16] and it was performed on the original data set consisting of 128 patients treated at University Medical Center Maribor.

The validation started with a binary analysis of proposed risk factors (Table 2). Four out five parameters used in the scoring by Halls et al. [26] were associated with IOC, but one could not be adequately analyzed – only two patients had previous open liver resection.

Then, points have been assigned to the proposed five risk factors related to a patient, disease, and surgery. The DSs were calculated and classified into four proposed levels (Table 3) [26]. Since the variable of previous liver resection added the highest score in the ranking into four different difficulty levels [26], the lack of patients in the extremely high difficulty group became evident.

The rates of IOC (0, 9.5, 55.5 and 100%) increased gradually with statistically significant values among difficulty levels (*P* < 0.001). This study additionally validated DSS by Halls et al. with various surrogates of technical operative difficulty. The difficulty level related well with transfusion requirements, operative time, the need for the hepatic pedicle clamping and its duration, and postoperative major morbidity (*P*-value among difficulty levels < 0.05) (Table 3).

However, the human mind tends to simplify matters and is not yet adapted to multidimensional reasoning, therefore artificial neural networks were developed [33, 34]. They are non-linear statistical data modeling tools [33, 34]. As such, they can be used to model complex, highly non-linear relationships between input and output variables of the observed phenomena [34, 37].

Using the same five independent variables (parameters) as introduced in the DSS by Halls et al. [26], a linear and nonlinear multivariate model were considered. Their correlation coefficients were 0.914 and 0.948, respectively. They represent high correlation between the validated DSS [26] and proposed linear and nonlinear multivariate models.

The validated DSS [26] has suggested four difficulty levels predicting the likelihood of IOC [26]. Based on the data originating from our center, a CDF representing the conditional (according to the validated DSS) probability of IOC during LLR was introduced. The mathematical background of the proposed CDF is based on the Weibull distribution which is used to model a variety of life behaviors [35]. Assuming the surgeon is experienced [10, 11], proposed CDF and a mean risk curve of IOC represent an objective risk estimation of an LLR at present (Fig. 3).

Currently, our center can perform LLR with an acceptable rate of IOC and postoperative morbidity when the patients are stratified in the low, moderate or high difficulty levels. On the contrary, LLR is still associated with obstacles and challenges for the extremely high-risk group of patients. When defining the mean risk curve, we assumed the risk for IOC to be equal to 1 for DS higher than 10. We believe that the assumption is justified as both patients with the highest DC had significant IOC.

Notably, the shape of the risk curve is defined based on all [26] and not just local data [35]. Therefore, we cannot expect any significant changes in the shape of the curve in the case of new data. Although two patients with DS = 10 experienced IOC, the mean risk curve predicts the probability of risk with 90%. Theoretically, this means that at least one in ten patients with DS = 10 would not experience any IOC during the LLR. Is there still enough room for improving the mean risk curve of IOC? Is it possible to perform liver surgery (laparoscopic or open) without any complications, especially as the limits of resectability are continually being pushed? Unlikely, but our main goal should be reducing rates of IOC and postoperative morbidity not only in the extremely high but in all risk groups.

Several implications of the proposed mean risk curve of IOC are possible. First, surgeons may familiarize themselves before surgery with an objective risk for IOC. Regarding the patient’s DS, the value in the proposed mean curve can serve as objective assistance in deciding on the type of liver surgery (laparoscopic or open). Secondly, the surgeon can objectively explain the risks of a surgical procedure and provide the patient with the risk probability of IOC. Thirdly, the hospital management can estimate the rate of expected IOC and related costs based on the CDF of patients. Fourth, the higher risk might be expected for surgeons just starting with these procedures and the steep learning curve of LLR should be recognized [10–12]. However, results can be always improved with specific training and mentoring [10, 11].

To the best of our knowledge, this study is the first external validation of the DSS proposed by Halls et al. done by an application of an artificial neural network. However, this study has some limitations associated with its retrospective nature. Another limitation is the low rate (1.6%) of LLRs in the extremely high difficulty group. This stresses the precise selection of patients considered for the laparoscopic approach, but the small sample size has statistical disadvantages. Moreover, the study is built on one surgeon’s procedures solely. It concomitantly increases the quality of the statements and decreases the statistical significance due to the number of the analyzed cases. Furthermore, our data were collected at a big academic center and may not reflect the risk of complications when surgeons perform LLR in smaller hospitals. Nonetheless, in our conviction LLR can develop only within the regular practice of liver surgery in high-volume centers.

## Conclusions

This external validation proved this DSS [26], based on patient’s, tumor, and surgical factors, enables us to estimate the risk of intra- and postoperative complications. The DSS was not only externally validated but upgraded with the proposition of the mean risk probability curve of IOC. It enables unprejudiced estimation of the probability of IOC considering the patients’ DS. Such objective information is of paramount importance for the patient, the surgeon, and hospital management as well. A surgeon should be aware of an increased risk of complications before starting with more complex procedures. To enhance skills safely, surgeons should start performing low difficulty procedures and gradually approach LLRs of higher difficulty.

## Data Availability

The data sets generated and/or analyzed during the current study are not publicly available due to the data is confidential patient data but are available from the corresponding author on reasonable request.

## Abbreviations

ANOVA: Analysis of variance
ASA: American Society of Anesthesiologists Score
CAE ANN: Conditional average estimator artificial neural network
CDF: Cumulative distribution function
DS: Difficulty score
DSS: Difficulty scoring system
IOC: Intraoperative complication
LLR: Laparoscopic liver resection

## References

[1] Reich H, McGlynn F, DeCaprio J, Budin R (1991). Laparoscopic excision of benign liver lesions. Obstet Gynecol.

[2] Ishizawa T, Gumbs AA, Kokudo N, Gayet B (2012). Laparoscopic segmentectomy of the liver: from segment I to VIII. Ann Surg.

[3] Soubrane O, Perdigao Cotta F, Scatton O (2013). Pure laparoscopic right hepatectomy in a living donor. Am J Transplant.

[4] Buell JF, Cherqui D, Geller DA, O'Rourke N, Iannitti D, Dagher I (2009). The international position on laparoscopic liver surgery: the Louisville statement, 2008. Ann Surg.

[5] Wakabayashi G, Cherqui D, Geller DA, Buell JF, Kaneko H, Han HS (2015). Recommendations for laparoscopic liver resection: a report from the second international consensus conference held in Morioka. Ann Surg.

[6] Ciria R, Cherqui D, Geller DA, Briceno J, Wakabayashi G (2016). Comparative short-term benefits of laparoscopic liver resection: 9000 cases and climbing. Ann Surg.

[7] Wong-Lun-Hing EM, van Dam RM, van Breukelen GJ, Tanis PJ, Ratti F, van Hillegersberg R (2017). Randomized clinical trial of open versus laparoscopic left lateral hepatic sectionectomy within an enhanced recovery after surgery programme (ORANGE II study). Br J Surg.

[8] Fretland ÅA, Dagenborg VJ, Bjørnelv GMW, Kazaryan AM, Kristiansen R, Fagerland MW (2018). Laparoscopic versus open resection for colorectal liver metastases: the OSLO-COMET randomized controlled trial. Ann Surg.

[9] Farges O, Goutte N, Dokmak S, Bendersky N, Falissard B (2014). How surgical technology translates into practice: the model of laparoscopic liver resections performed in France. Ann Surg.

[10] van der Poel MJ, Besselink MG, Cipriani F, Armstrong T, Takhar AS, van Dieren S (2016). Outcome and learning curve in 159 consecutive patients undergoing total laparoscopic hemihepatectomy. JAMA Surg.

[11] Villani V, Bohnen JD, Torabi R, Sabbatino F, Chang DC, Ferrone CR (2016). “Idealized” vs. “true” learning curves: the case of laparoscopic liver resection. HPB (Oxford).

[12] Abu Hilal M, Aldrighetti L, Dagher I, Edwin B, Troisi RI, Alikhanov R (2018). The Southampton consensus guidelines for laparoscopic liver surgery: from indication to implementation. Ann Surg.

[13] Ban D, Tanabe M, Ito H, Otsuka Y, Nitta H, Abe Y (2014). A novel difficulty scoring system for laparoscopic liver resection. J Hepatobiliary Pancreat Sci.

[14] Hasegawa Y, Wakabayashi G, Nitta H, Takahara T, Katagaru H, Umemura A (2017). A novel model for prediction of pure laparoscopic liver resection surgical difficulty. Surg Endosc.

[15] Kawaguchi Y, Fuks D, Kokudo N, Gayet B (2018). Difficulty of laparoscopic liver resection: proposal for a new classification. Ann Surg.

[16] Hallet J, Pessaux P, Beyfuss KA, Jayaraman S, Serrano PE, Martel G (2019). Critical appraisal of predictive tools to assess the difficulty of laparoscopic liver resection: a systematic review. Surg Endosc.

[17] Uchida H, Iwashita Y, Saga K, Takayama H, Watanabe K, Endo Y (2016). Clinical utility of the difficulty scoring system for predicting surgical time of laparoscopic liver resection. J Laparoendosc Adv Surg Tech.

[18] Im C, Cho JY, Han HS, Yoon Y-S, Choi Y, Jang JY (2017). Validation of difficulty scoring system for laparoscopic liver resection in patients who underwent laparoscopic left lateral sectionectomy. Surg Endosc.

[19] Tanaka S, Kubo S, Kanazawa A, Takeda Y, Hirokawa F, Nitta H (2017). Validation of a difficulty scoring system for laparoscopic liver resection: a multicenter analysis by the endoscopic liver surgery study Group in Japan. J Am Coll Surg.

[20] Periyasamy M, Cho JY, Ahn S, Han HS, Yoon Y-S, Choi Y (2017). Prediction of surgical outcomes of laparoscopic liver resections for hepatocellular carcinoma by defining surgical difficulty. Surg Endosc.

[21] Lee SY, Goh BKP, Sepideh G, Allen JC, Merkow RP, Teo JY (2019). Laparoscopic liver resection difficulty score – a validation study. J Gastrointest Surg.

[22] Uchida H, Iwashita Y, Tada K, Saga K, Takayama H, Hirashita T (2018). Laparoscopic liver resection in cirrhotic patients with specific reference to a difficulty scoring system. Langenbeck's Arch Surg.

[23] Yang J, Yang Z, Jia G, Xi Y, Xu Y, Li P (2019). Clinical practicality study of the difficulty scoring systems DSS-B and DSS-ER in laparoscopic liver resection. J Laparoendosc Adv Surg Tech A.

[24] Tanaka S, Kawaguchi Y, Kubo S, Kanazawa A, Takeda Y, Hirokawa F (2018). Validation of index-based IWATE criteria as an improved difficulty scoring system for laparoscopic liver resection. Surgery..

[25] Krenzien F, Wabitsch S, Haber P, Kamali C, Brunnbauer P, Benzing C (2018). Validity of the Iwate criteria for patients with hepatocellular carcinoma undergoing minimally invasive liver resection. J Hepatobiliary Pancreat Sci..

[26] Halls MC, Berardi G, Cipriani F, Barkhatov L, Lainas P, Harris S (2018). Development and validation of a difficulty score to predict intraoperative complications during laparoscopic liver resection. Br J Surg.

[27] Ivanecz A, Krebs B, Stozer A, Jagric T, Plahuta I, Potrc S (2017). Simultaneous pure laparoscopic resection of primary colorectal cancer and synchronous liver metastases: a single institution experience with propensity score matching analysis. Radiol Oncol.

[28] Ivanecz A, Pivec V, Ilijevec B, Rudolf S, Potrč S (2018). Laparoscopic anatomical liver resection after complex blunt liver trauma: a case report. Surg Case Rep.

[29] Moris D, Tsilimigras DI, Machairas N, Merath K, Cerullo M, Hasemaki N (2019). Laparoscopic synchronous resection of colorectal cancer and liver metastases: a systematic review. J Surg Oncol.

[30] Pugh RN, Murray-Lyon IM, Dawson JL, Pietroni MC, Williams R (1973). Transection of the oesophagus for bleeding oesophageal varices. Br J Surg.

[31] Strasberg SM, Belghiti J, Clavien PA, Gadzijev E, Garden JO, Lau WY (2000). The Brisbane 2000 terminology of liver anatomy and resections. HPB..

[32] Clavien PA, Barkun J, de Oliveira ML, Vauhey JN, Dindo D, Schulick RD (2009). The Clavien-Dindo classification of surgical complications: five-year experience. Ann Surg.

[33] Grabec I, Sachse W (2009). Synergetics of measurement, prediction and control.

[34] Peruš I, Poljanšek K, Fajfar P. Flexural deformation capacity of rectangular RC columns determined by the CAE method. Eartq Eng Struct D. 2006. 10.1002/eqe.584.

[35] Weibull W (1951). A statistical distribution function of wide applicability. J Appl Mech.

[36] Simard R, L’Ecuyer P (2011). Computing the two-sided Kolmogorov-Smirnov distribution. J Stat Softw.

[37] Terčelj M, Peruš I, Turk R. Suitability of CAE neural networks and FEM for prediction of wear on die radii inhot forging. Tribol Inter. 2003. 10.1016/S0301-679X(02)00246-3.

